# Supplementary material for: Association of viral kinetics, infection history, NS1 protein with plasma leakage among Indonesian dengue infected patients
Source: PLoS One. 2023 May 2;18(5):e0285087. doi: 10.1371/journal.pone.0285087 (PMC10153689; doi:10.1371/journal.pone.0285087)
Supplement: Attachment — Submitted filename: Review_FT-final.docx [file pone.0285087.s002.docx]

In the manuscript PONE-D-21-31549, entitled “Association of Viral Kinetics, Infection History and Plasma Leakage among Indonesian Dengue Infected Patients” authors describe a small prospective observational study including 48 cases of dengue. The manuscript brings relevant information, although not new, that viral load is correlated with severity (plasma leakage/DHF) specially in primary dengue. The manuscript could be suitable for publication once small modifications are done.

-Page 4, line 107: Authors should better describe the methodology and modifications of Lanciotti’s two-step reverse transcriptase PCR used to determine serotype. Which primers were used?

-Page 6: What is the asterisk in Table 1?

-Replace the term copy/mL for copies/mL

-Table 3 legend should briefly describe method used for serotyping and statistical analysis.

-Authors should briefly describe the dengue grades classification based on the 1997 WHO classification and include number of participants of each subgroup. Tables should differentiate hemorrhagic manifestation withing the groups as well as DHF I and II. All DHF patients displayed plasma leakage signs were classified as DHF since day 1? Table 3 should also include DF, DHF-DSS classification.

-Authors should standardize group names: at some parts of the manuscript groups are called “with plasma leakage” and “without plasma leakage” and in other parts of the text/figures groups are called “plasma leakage” and “no plasma leakage” or non-plasma leakage.

Some sentences are misleading, see examples below:

-Page 7, Lines 170-171 “Between patients with and without plasma leakage, the viral load was significantly different on day 4 (P=0.010) of fever.” Should be rewritten to be more specific:

Suggestion: “When comparing patients under primary infection with versus without plasma leakage, the viral load was significantly higher on day 4 (P=0.010) of fever in the leakage group.”

-Page 7, Lines 170-173: “In patients with secondary infection, the viral load levels of plasma leakage patients were also higher compared to patients without plasma leakage especially on day 1, 3, and 4. However, we did not find a significant difference of viral load in patients with secondary infection (Figure 2B).”

Suggestion: “In patients with secondary infection, the viral load levels of plasma leakage patients were also higher compared to patients without plasma leakage especially on days 1, 3, and 4 although no significant differences were found (Figure 2B).”

-Page 9, line 211. Authors mentioned one case of coinfection in the study, however it is not clear whether the co-infection case was included in the data since table 2 show 28 cases of no leak and 20 cases of leak.

-Language must be revised throughout the text. Some sentences are broken and need to be rewritten. See example below:

Page 10, Lines236-237: “While this may imply more rapid viral clearance in secondary infection, further investigation is still needed as a different marker compared to our study is used.”
