# Supplementary material for: Association of viral kinetics, infection history, NS1 protein with plasma leakage among Indonesian dengue infected patients
Source: PLoS One. 2023 May 2;18(5):e0285087. doi: 10.1371/journal.pone.0285087 (PMC10153689; doi:10.1371/journal.pone.0285087)
Supplement: Attachment — Submitted filename: Rebuttal Letter PLOS One 1304.docx [file pone.0285087.s004.docx]

**Response to Reviewer**

The technical detail that you have added in your revision is satisfactory, however the writing needs further revision to resolve lack of clarity. Please be sure to have your manuscript edited by a native speaker of the English language who is a scientist. If necessary, you might employ a scientific editing service.

**Answer:** Respected reviewers and editors, thank you for your inputs. We have reviewed our manuscript to improve the writing and have requested a native speaker to review our manuscript. May you find it satisfactory for publication in your journal.

Please review your reference list to ensure that it is complete and correct. If you have cited papers that have been retracted, please include the rationale for doing so in the manuscript text, or remove these references and replace them with relevant current references. Any changes to the reference list should be mentioned in the rebuttal letter that accompanies your revised manuscript. If you need to cite a retracted article, indicate the article’s retracted status in the References list and also include a citation and full reference for the retraction notice.

**Answer:** Thank you for the input. We have reviewed our reference list and made some corrections. We have not found any retracted references however some adjustments to the reference list were made as follows:

- All DOI identifiers, database’s unique identifiers have been removed to comply to samples of standard journal article provided by International Committee of Medical Journal Editors (ICMJE)
- Reference number 2: we have changed Asia WROFS-E to World Health Organization
